# Discovery Research on the Success Factors for Schools‐Based Multiple Model Speech and Language Placements: A Rapid Scoping Review

**DOI:** 10.1111/1460-6984.70331

**Published:** 2026-09-10

**Authors:** Despina Laparidou, Sophie Willis, Louise Howe, Aparna Ramachandran

**Affiliations:** ^1^ University of Lincoln Lincoln UK

**Keywords:** clinical supervision models, rural and underserved communities, school‐based placements, service‐learning in education, workforce development

## Abstract

**Background:**

Demand for children's speech and language therapy (SLT) services has surged globally, exacerbated by the COVID‐19 pandemic and workforce shortages. In England, waiting lists remain extensive, with over 66 000 children awaiting intervention in early 2025. Delayed access to SLT services is linked to poorer academic, social, and health outcomes. Innovative approaches are urgently needed to address these challenges. Embedding SLT student placements within schools offers a potential solution, simultaneously enhancing service capacity and providing authentic learning experiences. International evidence, such as the Broken Hill model in Australia, supports this approach, particularly for rural and remote communities.

**Aims:**

This rapid scoping review aimed to identify success factors, barriers, and enablers of school‐based SLT placement models, and to assess their potential for scalability and sustainability. The review sought to inform policy and practice by synthesising evidence on supervision models and implementation strategies.

**Methods:**

Following PRISMA‐ScR guidelines and Arksey & O'Malley's framework, a comprehensive search was conducted across eight databases (e.g., Web of Science, ERIC, CINAHL, Medline) and supplemented by grey literature searches. Inclusion criteria focused on SLT student placements in schools, particularly in underserved areas. Fourteen studies published between 2011 and 2024 were included, predominantly from Australia (57%), with others from the USA and UK. Data were extracted and synthesised narratively around barriers, facilitators, and model characteristics.

**Main Contribution:**

Findings indicate that school‐based SLT placements can increase service capacity, improve student confidence and employability, and foster interprofessional collaboration. Successful models share key principles: Integration into policy frameworks, dedicated funding, strong cross‐sector partnerships, cultural responsiveness, and structured supervision. Barriers include insufficient funding, infrastructure limitations in rural areas, student preparedness, and challenges with technology during remote delivery. Facilitators include early stakeholder engagement, flexible supervision approaches, and orientation training. Evidence suggests that positive placement experiences may influence graduates’ willingness to work in underserved areas, addressing workforce shortages.

**Conclusions:**

School‐based SLT placements represent a promising strategy to alleviate service pressures and enhance workforce development. Greater alignment with health and education policy, sustainable funding, and approaches tailored to diverse contexts may support successful implementation. Scaling effective models could reduce waiting lists, improve equitable access to SLT services, and provide high‐quality experiential learning for future practitioners.

**WHAT THIS PAPER ADDS:**

*What is already known on this subject*

- School‐based SLT placements have been implemented internationally, particularly in rural and underserved areas, to address workforce shortages and improve access to services. Evidence suggests these models can enhance student learning and service capacity, but there is limited synthesis of success factors, barriers, and scalability in the UK context.

*What this study adds to the existing knowledge*

- This review identifies key principles for successful school‐based SLT placements, including integration into policy frameworks, dedicated funding, cross‐sector partnerships, and cultural responsiveness. It highlights barriers such as infrastructure limitations and student preparedness, and demonstrates that positive placement experiences may influence future workforce distribution. These findings provide an evidence‐informed foundation for scaling models to reduce waiting lists and improve equitable access.

*What are the clinical implications of this study?*

- Embedding SLT student placements in schools can increase service capacity, reduce waiting times, and support early intervention for children with speech and language needs. Structured supervision and orientation, alongside policy alignment and appropriate funding, may support sustainability and workforce readiness across diverse clinical contexts.

## 1 Background

In recent years, there has been an increased demand for children's speech and language therapy (SLT) services. This situation has been made worse by the Covid‐19 pandemic, with many SLT services facing continuous and significant challenges as they try to meet existing needs and demands for services with growing referrals and waiting lists for children to receive SLT provision (Department of Education 2022; Royal College of Speech and Language Therapists 2022). For example, the number of children and young people (CYP) with speech, language and communication needs has increased by 42% in the last decade (Royal College of Speech and Language Therapists 2023). Similar increases in demand have been documented not only in the United Kingdom (UK), but elsewhere in the world as well, including Ireland (Müller et al. 2024) and Australia (Marozzi 2022; Smith et al. 2021).

As a result, waiting lists for SLT are common, particularly in countries with government subsidised health care such as the UK. In the UK, data showed that over 65 600, in 2022, and 67 774, in 2023, CYP were waiting for SLT (NHS Confederation 2022; NHS England 2023). Although more recent data show a slight decrease in numbers, there were still 66 612 CYP waiting to receive speech & language therapy interventions in January 2025 (NHS England 2025). Out of these 66 612 CYP, almost 21 000 had been on a waiting list for more than 18 weeks (NHS England 2025).

Long waiting lists and delayed access to SLT services can lead to poorer academic and behavioural outcomes (Children's Commissioner for England 2019; Conway et al. 2021; Royal College of Speech and Language Therapists 2023; Smith et al. 2021), as well as physical and mental health problems (Botting et al. 2016; Hancock et al. 2023; Speech and Language UK 2023), communication and socialising issues (Bercow 2008; Cohen et al. 2013; Conway et al. 2021; Hitchcock et al. 2015; Public Health England 2020), and even financial difficulties for the children's family and their own future (New Zealand Speech‑language Therapists’ Association 2024; Speech Pathology Australia 2023).

There is an urgent need to consider how SLT placements are delivered in England, particularly in the context of growing demand, workforce shortages, and widening inequalities in access to services. SLT students undertake practice‐based learning across a range of settings and through different models of supervision. In light of these challenges, and the fact that no other relevant reviews exist currently, this scoping review was commissioned to explore schools‐based multiple model SLT placements as an alternative approach to placement delivery. Embedding SLT students into schools offers a potential win‐win: enabling high‐quality, hands‐on student learning while increasing SLT service capacity in the education sector. International evidence, such as the Broken Hill model in Australia, supports the viability of this approach, particularly for rural and remote communities (Jones et al. 2011).

This rapid scoping review aims to assess the characteristics, barriers, and enablers of these models and to provide an evidence‐informed foundation for scalable and sustainable placement reform. In doing so, it contributes to ongoing efforts to improve children's access to SLT services, support the development of the future workforce, and reduce systemic inequalities.

## 2 Methods

We followed the Preferred Reporting Items for Systematic reviews and Meta‐Analyses extension for Scoping Reviews (PRISMA‐ScR) Checklist (Tricco et al. 2018) and the Arksey and O'Malley framework (2005) for conducting this scoping review. The review protocol was registered in Open Science Framework (osf.io/9fmge).

The review team brought together expertise in SLT, qualitative research and allied health professional education and research. AP is a speech and language therapist, DL is a qualitative researcher, and SW has expertise in allied health professional education and research. Individual roles in study selection, data extraction and checking are described below.

### 2.1 Inclusion Criteria

Our inclusion criteria were: Students on SLT placements in schools (Participants); Community‐based school SLT placements in which students are deployed within the workforce to support high quality practice‐based learning and increase access for children to first line assessment and treatment within multiple models of clinical supervision (Concept); Community‐based schools, especially those in underserved areas (e.g., rural, coastal, high‐inequality regions) (Context).

### 2.2 Types of Sources

To ensure no relevant evidence was excluded, the review considered a comprehensive range of evidence sources, including experimental, quasi‐experimental, observational, and qualitative study designs. In recognition of the exploratory nature of scoping reviews, opinion papers were considered in order to capture expert perspectives and contextual insights not readily available through empirical research. A formal critical appraisal of included sources was not undertaken, as the purpose of the review was to map the breadth and characteristics of the available evidence rather than to determine intervention effectiveness. This also enabled evidence from a range of study designs and sources to be considered in addressing the review aims.

### 2.3 Search Strategy

The search strategy aimed to locate both published and unpublished studies with identified keywords and index terms adapted for each database and/or information source. The reference list of any systematic reviews on the same/similar topic were screened for additional studies. Only studies published in English were included, due to limited resources. For the full search strategy used for all databases via EBSCOhost, see Appendix 1.

The databases/search engines that were searched (from 2015 to January 2025) included Web of Science core collection and, via EBSCOhost, ERIC (Education Resources Information Center), Educational Administration Abstracts, British Education Index, Education Source, CINAHL Ultimate, AMED and Medline. PROSPERO was also searched for protocols of existing (completed or ongoing) systematic reviews. Finally, all database searches were supplemented with internet searches as well (i.e., Google Scholar). The full search strategy for all databases is provided in Appendix 1.

### 2.4 Study/Source of Evidence Selection

**FIGURE 1:**
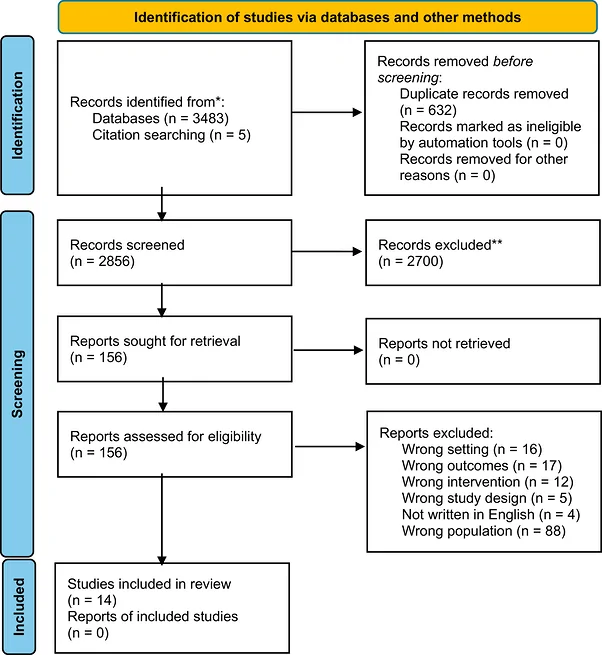
PRISMA flow diagram of study selection.

Following the search, all identified citations were collated and uploaded into Covidence and duplicates were removed. In keeping with the rapid approach to this scoping review, titles and abstracts were screened by DL against the predetermined inclusion criteria. Potentially relevant sources were retrieved in full and assessed in detail against the same inclusion criteria by DL. Covidence was used to support consistent management of the screening process, and reasons for exclusion at full text were recorded in a PRISMA flowchart (see Figure 1) (Page et al. 2021). Any uncertainty at either stage of the selection process was discussed and resolved among all reviewers (DL, AP, SW).

### 2.5 Data Extraction

Data were extracted from the included papers by DL, using a data extraction tool developed by the reviewers, with a second reviewer (AP) checking 20% for accuracy. Any discrepancies were resolved through discussion.

Extracted information included: study details (title, authors, date, country), methods (aims, study design, setting, data collection methods, placement model characteristics), participant characteristics (sample size and demographics), and study findings (outcomes, main findings, barriers and facilitators).

### 2.6 Data Analysis and Presentation

A narrative synthesis of all studies was performed and structured around the review's objectives (i.e., barriers and facilitators for schools‐based multiple supervision models for SLT placements; any existing models, including any international models; etc.).

## 3 Results

The search strategy identified a total of 3488 citations and, after removing duplicates, title/abstract and full text screening, 14 studies were included in this rapid scoping review. Figure 1 presents a PRISMA flowchart (Page et al. 2021) illustrating the results of the selection process.

### 3.1 Study Characteristics

The 14 included studies were published between 2011 and 2024 and were mainly from Australia (*n* = 8; 57.1%), with 4 studies (28.6%) being conducted in the United States of America (USA) and 2 (14.3%) in the United Kingdom (UK). Many studies (*n* = 8; 57.1%) were conducted only in rural areas (*Dodd et al. 2019; Jones et al. 2011; *Jones et al. 2015, 2015b; Jones et al. 2015c; Jones et al. 2016; *Kirby et al. 2018; *Moran et al. 2024), two studies (14.3%) were conducted in both urban and rural areas (Allison and Thompson 2023; Bowers et al. 2022), and one study (7.1%) was conducted in an urban environment (Koutsoftas et al. 2024). Finally, two papers (14.3%) did not specify the type of environment (*Giess et al. 2020; Hopkins et al. 2022) and one study (7.1%) was a text and opinion paper (Marante and Hall‐Mills 2019).

### 3.2 Narrative Synthesis

#### 3.2.1 Supervision Models

Twelve papers (85.7%) discussed supervision models for student schools‐based placements. The most used supervision model (implemented by 50% of the studies) was the multidisciplinary rural clinical placement program in far western New South Wales, Australia, operated by the Broken Hill University Department of Rural Health (Jones et al. 2011; *Jones et al. 2015, 2015b; Jones et al. 2015c; Jones et al. 2016; *Kirby et al. 2018).

The Broken Hill program was piloted in 2009 and has been successfully evolved and implemented in various sites throughout the years.

The current Broken Hill model (*Jones et al. 2015; Jones et al. 2015b; Jones et al. 2015c, 2016; *Kirby et al. 2018) involves four occupational therapy (OT) and six SLT students undertaking placements across four school terms. Data from interviews and focus groups showed that ‘student participation in the program enhanced continuity of care and capacity to practice inter‐professionally’ (Jones et al. 2015b). The main ‘factors supporting partnerships were long‐term, work and social relationships, commitment to community, trust and an appetite for risk‐taking’ (*Kirby et al. 2018). Students also ‘reported enhanced perceptions of future employability through “real work” experiences’ (Jones et al. 2015c).

Allison and Thompson (2023) used peer‐assisted learning (PAL) in a group model of student placement to enable student‐led service delivery. This model involved both group‐based coaching and one‐to‐one supervision, where eight students, working in pairs, completed an 8‐week block placement of 4 days a week and an additional half a day of self‐directed study. Their findings showed that students felt more confident in all clinical areas, the schools reported high levels of satisfaction and, the children achieved 60% of the targets set by the SLT students.

Bowers et al. (2022) explored the preparation of speech‐language pathology and elementary education students during their educational practicum settings during the COVID‐19 pandemic. SLT students spent 10 h each week at their school site, including a minimum of five direct patient/client contact hours per week. Despite significant challenges due to the pandemic, findings showed that both students and their mentors adapted adequately and were able to navigate their field experiences successfully.

In 2019, Dodd and colleagues discussed their evaluation of a clinical placement model, during which eight second‐year SLT Masters students provide a 4‐week intensive student clinic each year. This model was also successful, with 83% of the children not needing ongoing SLT support.

Another study (Hopkins et al. 2022) involved students as part of a practice‐based research placement, where the Word Aware 2 (WA) intervention was implemented. WA is a structured whole‐school language intervention. The placement lasted 10 weeks, during which the students supported the teaching staff in the delivery of all aspects of the WA STAR approach. Although there were no statistically significant differences between the intervention and control groups in overall improvement, there was significant improvement within each group on words targeted for intervention, positive observations of child engagement and students’ increased sense of confidence in their ability to collaborate with teaching staff.

In a more recent paper, Koutsoftas and colleagues (2024) presented the results of their 3‐year program evaluation of a collaborative classroom‐based intervention, where graduate student interns were placed in school settings. Students observed and participated in speech therapy sessions of children with the speech and language pathologist of the school and engaged with children in their classrooms, during recess, at specials (i.e., music, gym, art), and at lunch. The program received positive feedback from all stakeholders and there was an increase in the number of classroom‐based intervention hours for school children receiving speech‐language services.

Finally, a text and opinion paper (Marante and Hall‐Mills 2019) presented their standards‐driven, workforce‐focused model of school practicum, which was based on the school practicum program offered in the School of Communication Science and Disorders at Florida State University. They propose what school‐based practicum curricula should include, such as appropriate standards for graduate education, scope of practice, collaboration with important stakeholders, evidence‐based practice. They recommend that, for students to have a holistic experience with sufficient breadth and depth, a minimum of 2 full school days weekly is required for a full university semester. Students should be, under the direction of their supervisor, working on service delivery, including face‐to‐face direct therapy services, direct nontherapy services and indirect services.

For a detailed description of the characteristics of supervision models, including frequency and format of supervision, student–supervisor ratios and reported outcomes, see Appendix 2.

#### 3.2.2 Characteristics of Supervision Models

Frequency and Duration of supervisory sessions: Five out of 12 papers (41.7%) gave no information regarding the frequency and duration of supervisory sessions for students (Bowers et al. 2022; Jones et al. 2011, 2016; *Kirby et al. 2018; *Moran et al. 2024). Two studies (16.7%) only mentioned how students were closely monitored by their placement supervisors (Word Aware 2 intervention, Hopkins et al. 2022; Marante and Hall‐Mills 2019), while others varied in the frequency of their supervisory sessions, ranging from once a week (*Jones et al. 2015; Jones et al. 2015b; Jones et al. 2015c) to twice a week (*Dodd et al. 2019). Other studies offered more complex models of group and paired weekly meetings, as well as individual fortnightly supervisions with the lead educator (Allison and Thompson 2023) or even a combination of three meetings per day between students and supervisors and weekly onsite supervision/support (Koutsoftas et al. 2024). All models also offered additional support when needed.

Types of sessions: Regarding individual or group settings, only two studies (16.7%) specified that they used both individual and group supervisory meetings (Allison and Thompson 2023; *Dodd et al. 2019). Four studies (33.3%) also involved peer supervisions or peer support and learning among students (Allison and Thompson 2023; *Jones et al. 2015; Jones et al. 2015b; Jones et al. 2015c). Nine studies (75%) did not specify how supervisions were conducted (Allison and Thompson 2023; Hopkins et al. 2022; Jones et al. 2011; *Jones et al. 2015, 2015b; Jones et al. 2016; *Kirby et al. 2018; Marante and Hall‐Mills 2019; *Moran et al. 2024). One study (8.3%) clearly stated that all supervisory sessions were conducted in person (*Dodd et al. 2019), whereas another study (Jones et al. 2015c) used a combination of face‐to‐face, email, SMS and telephone communications. One study (Bowers et al. 2022) was conducted during Covid‐19, so all classes and meetings with academic faculty were conducted virtually. Finally, one study (Koutsoftas et al. 2024) conducted face‐to‐face supervisions initially, but had to switch to remote instruction, due to the Covid‐19 pandemic.

Student to supervisor ratios: Out of 12 papers only four studies (33.3%) provided information regarding student to supervisor ratio. Allison and Thompson (2023), identified that students were shared, in pairs, between 3 lead educators. In another study (*Dodd et al. 2019), two supervisors supervised 8 students, whereas the Jones and colleagues (2015b) paper described having a 6:1 student to supervisor ratio. Finally, another study (Koutsoftas et al. 2024), reported that each supervising speech and language pathologist was assigned two to three graduate students.

#### 3.2.3 Barriers to the Implementation and Success of Supervision Models

Despite the mostly positive feedback and outcomes of the implementation of the above placement models, researchers also noted various challenges and barriers to such models.

Policies, funding and resources: Three papers (21.4%) discussed the negative impact of lack of funding and infrastructure particularly in underserved populations (*Jones et al. 2015; *Kirby et al. 2018; *Moran et al. 2024). *Jones et al. (2015) identified the need to consider the individual characteristics of the region where a placement model takes place (such as their unique geography, population, health and education systems). Issues such as travel, staffing/human resources, accommodation costs and availability were all highlighted as potential barriers to successful implementation and/or sustainability of placement models in small, rural and remote communities (*Moran et al. 2024). Embedding the models into government health and education policy, thus, securing funding, was also considered vital (*Kirby et al. 2018).

The developing student practitioner: According to the study by *Giess et al. (2020), recent graduate clinicians and supervisors had different views regarding the skills and preparation necessary for students to be successful in their work. Supervisors believed that students were not well prepared to be able to handle the pupils’ problem behaviours, but this skill did not hold the same importance for graduate clinicians. On the contrary, students felt they were least prepared for the billing process, but the supervisors were less concerned about this skill.

Another point raised in the studies (Hopkins et al. 2022) was that students often initially felt overwhelmed and struggled with the responsibility of being part of the intervention and collaborating with the teaching staff. After working with the teaching staff in a more collaborative and proactive way, these feelings were replaced by feelings of trust in the teachers’ ability to maintain the delivery of the intervention (Hopkins et al. 2022). Similarly, Jones et al. (2015c) found that students struggled to transition from previous working experiences to a semi‐autonomous service‐learning role. Students seemed to struggle with decision making, case load management, development and implementation of therapy plans, preparation of documentation, preparing new or using existing resources, as well as having little to no formal prior inter‐professional practice experience during their training (Jones et al. 2015b; Jones et al. 2015c).

The paper by *Kirby et al. (2018) highlighted that support for students on placement was costly, with common issues being poor pre‐placement preparation, lack of cultural understanding, no ability to self‐manage and/or meet learning goals.

Finally, *Jones et al. (2015) stressed that sustainability of any placement model, especially in rural and remote areas, relies on the availability and accessibility of suitably qualified SLTs. Thus, the challenge for higher education institutions is to develop courses and provide opportunities for clinical placements that take into consideration the health needs and expectations of the communities they serve. Students who have positive experiences while on placement in such areas may be more likely to consider returning to these areas after graduating (*Jones et al. 2015).

Placements and technology: The study by Bowers et al. (2022) was conducted during the Covid‐19 pandemic and, therefore, was almost exclusively conducted virtually/online. Students felt that technology was a barrier to implementing therapy and keeping their pupils engaged online and that the learning support and feedback they needed from their supervisors was limited. Technology and tele practice also complicated building strong relationships and positive interactions among students, pupils, and supervisors.

Environment issues were discussed by *Kirby et al. (2018), who reported various school‐related problems, such as access to clinic space, withdrawing school pupils from class and new teachers settling into the program. These issues were resolved through negotiation and shared decision making between the involved parties, that is, the school and the host academic.

Rural versus urban locations: One study (Allison and Thompson 2023) highlighted the fact that students were allocated to placements in mainstream schools mostly and, therefore, worked with a limited population. This point was echoed by another study (Jones et al. 2015c) that discussed how the transferability of their placement model to other contexts is not well understood and further research is needed. Both studies posed the question whether their placement model would be equally appropriate for other clinical caseloads (such as children with more complex needs perhaps, adults and/or metropolitan contexts).

In addition, studies (Jones et al. 2016; *Moran et al. 2024) cautioned that working in rural/remote areas might not be suitable for all students and may even be a barrier to them accepting a placement in such areas and/or may lead to less‐than‐optimal learning outcomes (Jones et al. 2016).

People and communities: Three papers (21.4%) highlighted the importance of building strong relationships among all stakeholders, including the communities SLTs work in (*Jones et al. 2015; Jones et al. 2016; *Moran et al. 2024). *Jones et al. (2015) emphasised the importance of understanding cultural differences and providing health services that are culturally responsive. These findings were echoed by another study (*Moran et al. 2024), which discussed the challenges of working in remote communities, where there may be elements of distrust, and they argued that one of the biggest challenges to service continuity can be the frequent changeover of students.

Barriers for supervisors: The study by Jones and colleagues (2015b) highlighted how academic supervisors struggled with their own professional development associated with program participation and student supervision. The study by *Moran et al. (2024) also noted how implementing service learning placements can be a big commitment for supervisors and other staff.

## 4 Discussion

This rapid scoping review explored the success factors of schools‐based SLT placements. The findings of the review have shown that these placement models have been largely successful in increasing capacity in schools and have been well received by both students and supervisors, as well as the communities in which they were implemented.

The main success factors discussed were incorporating the placement programs into government policy, securing funding for them, as well as adequate resources (*Jones et al. 2015; *Kirby et al. 2018; *Moran et al. 2024); model partners need to be committed, flexible, and have the capacity to respond quickly to challenges (*Jones et al. 2015); building good relationships, teamwork, camaraderie, and collaboration among key stakeholders (Jones et al. 2015b; Jones et al. 2015c; *Kirby et al. 2018; Koutsoftas et al. 2024; *Moran et al. 2024), especially within the communities (*Jones et al. 2015; Jones et al. 2016; *Kirby et al. 2018); ensuring service continuity (*Jones et al. 2015; Jones et al. 2015b, 2016); and including an orientation component to the placements (Jones et al. 2015c).

Policies, funding and resources: Two studies (14.3%) highlighted the importance of securing funding (*Jones et al. 2015; *Kirby et al. 2018), while aligning placement models/interventions to governmental policies and community needs (*Jones et al. 2015). To implement supervision models successfully, studies recommended establishing early partnerships across health, school education and higher education sectors (*Jones et al. 2015); having stakeholders share resources to maintain the service learning programmes in the absence of government funding (*Kirby et al. 2018); as well as university staff visiting rural communities to help their commitment to preparing and sending students (*Moran et al. 2024).

Model characteristics: As a model matures and becomes integrated into daily practices, *Jones et al. (2015) argued that the model partners need to be committed, flexible, and have the capacity to respond quickly to challenges, so that the program can achieve long‐term positive outcomes and sustainability. More specifically, the model needs to be able to acknowledge unpredictable and unplanned phenomena, learn from being implemented in everyday practice and adapt accordingly (*Jones et al. 2015). This adaptability was particularly evident in how supervision was conceptualised and operationalised across models.

Across the studies reviewed, no single supervision model emerged as optimal in terms of frequency, format or student–supervisor ratio. Instead, effective supervision appeared to be characterised by flexibility, relational continuity and responsiveness to both student development and local service context. Models that incorporated shared supervision, peer‐assisted learning or mixed modes of contact were repeatedly associated with increased placement capacity and positive student experiences, even where formal supervisory structures were limited or inconsistently reported. This suggests that, within school‐based SLT placements, supervision functions less as a fixed set of activities and more as an enabling mechanism that supports autonomy, service integration and learning‐in‐practice, particularly in settings facing workforce or resource constraints.

The developing student practitioner: Five papers (35.7%) described how students valued their time in school placements, as they perceived this experience allowed them to develop essential knowledge and competencies (*Giess et al. 2020; Hopkins et al. 2022; Jones et al. 2015b; Jones et al. 2015c, 2016). Building good relationships, teamwork, camaraderie, collaboration among key stakeholders (students, supervisors, etc.), and holding regular debrief meetings were considered equally important (Jones et al. 2015c; *Kirby et al. 2018; Koutsoftas et al. 2024; *Moran et al. 2024), as was the supervision approach which students felt contributed to the development of higher levels of autonomy in professional and technical decision making (Jones et al. 2015b; Jones et al. 2015c, 2016).

When placements involved inter‐professional practice, for example the Jones and colleagues’ study (2015b), students valued the opportunity to learn more about collaboration, interprofessional integration of therapy goals or the exchange of information regarding successful intervention strategies (Jones et al. 2015b). A second study (Koutsoftas et al. 2024), proposed that teaching collaborative classroom‐based intervention approaches would be beneficial to students during their placements.

Finally, the study by Jones et al. (2015c) also identified that the opportunity to impart knowledge leads to a level of understanding required to adapt and apply their skills to more complex environments. Whilst *Moran et al. (2024), argued for the importance of students to receive comprehensive cultural sensitivity training to equip them for challenges of working with diverse communities.

Rural/urban locations: Students and supervisors valued the opportunity to work in rural communities (Jones et al. 2015c; Jones et al. 2016). Orientation training and preparation ensured that students understood the context of their placement and alternative health care approaches. According to Jones et al. (2015c), ‘understanding contexts of care that impact on community health is essential in the provision of population healthcare.’ Support and acculturation received, especially from the residents of the rural communities, and developing relationships with students from other professions was viewed positively and decreased anxiety related to being separated from their peers (Jones et al. 2015b, 2016).

People and communities: Three studies (21.4%) highlighted the importance of building trust and close relationships within the community (*Jones et al. 2015; Jones et al. 2016; *Kirby et al. 2018). Accordingly, the model partners need to be able to show consistency and credibility, so that they can build trust and instil confidence in the local populations where the placements are taking place. This is especially true in remote areas where people (often Indigenous populations) can be more guarded towards new programs and their longevity (*Jones et al. 2015; Jones et al. 2016). Furthermore, *Jones et al. (2015) suggested that being able to commit to long periods of implementation (at least a 7‐year program commitment) can enable partners to respond to various needs and crises as they arise, adapt accordingly and gain trust among the people they are serving.

Facilitators for supervisors: According to Bowers et al. (2022), utilising online supervision was convenient for supervisors, as it allowed them to provide support to students and communicate more frequently. *Kirby et al. (2018) highlighted the importance of supervisors, serving as troubleshooters and key channel of communication between students, host and source universities and schools. Finally, the study by *Moran et al. (2024) argued that supervisors needed to facilitate and manage a range of supervision approaches, be supportive and responsive to student and community needs, and create high‐quality learning opportunities for students.

Despite the positive feedback and outcomes associated with the implementation of the placement models, the review also revealed various challenges and barriers. The main identified barriers were the need for more funding, resources and infrastructure in underserved populations, (*Jones et al. 2015; *Kirby et al. 2018; *Moran et al. 2024); ensuring students have adequate training and education to develop competence within their role and the responsibility of working autonomously as an SLT practitioner in a school environment (*Giess et al. 2020; Hopkins et al. 2022; *Jones et al. 2015; Jones et al. 2015b; Jones et al. 2015c; *Kirby et al. 2018); utilising online technology, such as during a pandemic (Bowers et al. 2022); and, the need for better understanding of cultural differences to improve engagement with diverse populations, and provide health services that are culturally responsive (*Jones et al. 2015; *Moran et al. 2024).

## 5 Limitations

This is the first systematic review exploring the success factors of schools‐based SLT placements. Strict methodological guidelines for the conduct of this review were followed; however, it is a rapid review and, therefore, only one reviewer conducted title/abstract and full text screening of all references for assessment against the inclusion criteria for the review. This choice may have allowed some bias in the selection of the studies, which was somewhat mitigated by having any uncertainty regarding all decisions discussed among the team of researchers.

## 6 Implications for Policy and Practice

The findings of this review have several important implications for policy and practice:

1. **Integration into policy frameworks**

The findings suggest that greater alignment of schools‐based SLT placement models with national health and education policy may support sustainability, alignment with workforce development strategies and consistency across regions.

2. **Dedicated funding**

Identifying funding to support costs particularly in rural and underserved areas where infrastructure may be lacking

3. **Pre‐placement preparation**

Ensuring that students are prepared for the challenges of this particular workplace and cultural competency addressed as appropriate.

4. **Cross‐sector partnerships**

Strong collaboration is needed between universities, health services and schools to ensure access to resources and support.

5. **Scaling to address workforce shortages and inequalities**

Expanding successful models across regions to support the existing SLT workforce in alleviating waiting lists and improving access to services.

6. **Utilise technology for flexible supervision**

Hybrid supervision approaches can be utilised particularly to support students in rural and underserved areas lacking in infrastructure.

## 7 Conclusions

This rapid scoping review demonstrates that schools based SLT placements offer a promising solution to the growing demand for SLT services and workforce shortages. The evidence reviewed suggests that schools based placement models can enhance student learning, foster interprofessional collaboration and increase service capacity. Challenges include funding and infrastructure, and the findings suggest that greater alignment with policy frameworks and stronger cross‐sector partnerships may support implementation. Scaling successful models and tailoring them to diverse contexts may build capacity, increase equitable access to SLT for children, and provide valuable learning opportunities for future practitioners.

## Funding

This rapid scoping review has commissioned by NHS England to undertake discovery research on the success factors for schools based multiple model Speech and Language (SALT) placements but the study was conducted independently and has not been verified by NHS England. Grant number: 0008293‐1060.

## Conflicts of Interest

The article is derived from an unpublished report commissioned by NHS England to undertake discovery research on the success factors for schools based multiple model Speech and Language (SALT) placements but the study was conducted independently and has not been verified by NHS England. All other authors have no completing interest to declare.

## Data Availability

All data requests should be submitted to the corresponding author for consideration.

## Appendix 1 Search strategy for all databases via EBSCOhost

### 1.1

**Table:**

Search ID	Search terms	Field
1	Speech therapy	MH
2	Language therapy	MH
3	Research, Speech‐Language‐Hearing Therapy	MH (CINAHL)
4	“Speech and language therap*”	AB
5	SLT	AB
6	“Speech and language patholog*”	TX
7	SLP	AB
8	“Speech therap*”	AB
9	Speech‐Language pathology	MH
10	Speech‐Language pathology	MH (CINAHL)
11	“Speech language patholog*”	AB
12	“Speech patholog*”	AB
13	“Language therap*”	AB
14	“Speech and Language placement*”	TX
15	Model* N2 clinical supervision*	AB
16	“Speech and Language Therapy student*”	TX
17	“SLT student*”	TX
18	“SLP student*”	TX
19	“Speech and language service*”	TX
20	“(SLT or Speech and Language Therapy) practitioner*”	TX
21	“In‐service learning placement model*”	TX
22	“Community‐based school speech and language therap* placement*”	TX
23	1 OR 2 OR 3 OR 4 OR 5 OR 6 OR 7 OR 8 OR 9 OR 10 OR 11 OR 12 OR 13 OR 14 OR 15 OR 16 OR 17 OR 18 OR 19 OR 20 OR 21 OR 22	
24	Schools	DE (ERIC)
25	Schools	MH
26	“School system*”	AB
27	“Education* system*”	AB
28	“Education* setting*”	AB
29	“Education* institution*”	AB
30	“School‐based placement*”	TX
31	“School placement*”	TX
32	Student placement	DE (ERIC)
33	Student placement	MH (CINAHL)
34	“Student placement*”	AB
35	“Student practicum”	TX
36	“Student posting*”	TX
37	“Education placement*”	TX
38	“Practice‐based learning”	TX
39	“Supervision Model*”	TX
40	“Placement model*”	TX
41	Student SLT placement* in school*	TX
42	“Broken Hill Rural University Hub”	TX
43	“Placement structure*”	TX
44	“Innovative supervision strateg*”	TX
45	24 OR 25 OR 26 OR 27 OR 28 OR 29 OR 30 OR 31 OR 32 OR 33 OR 34 OR 35 OR 36 OR 37 OR 38 OR 39 OR 40 OR 41 OR 42 OR 43 OR 44	
46	23 AND 45	

## Appendix 2 Characteristics of supervision models

### 2.1

**Table:**

Supervision model	Frequency & duration of supervisory sessions	Types of sessions	Student to supervisor ratios	Outcomes of supervision models
Allison and Thompson 2023; UK	Weekly group supervision, at least once weekly paired supervision & individual supervisions with the lead educator fortnightly. Students also accessed informal peer supervision (focusing on problem‐solving, planning and reflection) throughout their placement, both within and outside of their pair.	Both individual and group supervisory meetings, as well as student peer supervisions	Students were shared between 3 lead educators, with one lead educator supporting two pairs of students, and the other lead educators supporting one pair of students	Students felt more confident in all clinical areas and working independently, an increase in the perceived benefit to the service, as well as that the schools reported high levels of satisfaction for working with these students. Finally, the children achieved 60% of the targets set by the speech and language therapy students.
Bowers et al. 2022; USA	No information reported	Took place during Covid‐19, so all classes/meetings were conducted virtually	No information reported	Despite significant challenges due to the pandemic, findings showed that both students and their mentors adapted adequately and were able to navigate their field experiences successfully.
*Dodd et al. 2019; Australia	Students were given an initial orientation and discussion of each referral, and case discussions with clinical educators after school at each site, 2 mornings a week (and when requested). Students also attended two seminars regarding differential diagnosis and treatment of speech sound disorders and literacy intervention.	In‐person individual and group supervisory meetings	Two supervisors supervised 8 students	83% of the children did not need ongoing speech and language therapy support.
*Giess et al. 2020; USA	Not applicable	Not applicable	Not applicable	Graduate clinicians and speech‐language pathology supervisors shared similar views regarding adequate preparation for a school‐based practicum, with both types of participants feeling that graduates received good or extensive preparation and were adequately or well prepared. Groups of participants differed in their expectations of what graduate students should know, skills identified as needing better development, and what is important for successful practice in the schools.
Hopkins et al. 2022; UK	Word Aware 2 (WA) intervention: students and teaching staff received training on model implementation and students were then closely monitored and guided on how to better support the delivery of the intervention by their placement supervisors. The research team also held an afternoon debrief session midway through the intervention to review progress and to offer solutions to any potential challenges students were facing.	No information reported	No information reported	Although there were no statistically significant differences between the intervention and control groups in overall improvement, there was significant improvement within each group on words targeted for intervention. Results also showed positive observations of child engagement, as well as students’ increased sense of confidence in their ability to collaborate with teaching staff.
Jones et al. 2011; Australia	No information reported	No information reported	No information reported	Feedback from everyone involved was positive and three of the students returned to work at Broken Hill after their placement was completed.
*Jones et al. 2015; Australia	Broken Hill model includes discipline‐specific, multidisciplinary academic and student peer supervisions, with teachers providing an additional layer of supervision for classroom activities. Students also have weekly clinical case discussions, where they can discuss therapeutic approaches and alternative methods of therapy delivery.	Type of sessions is not specified, but they include additional student peer supervisions	No information reported	Some of the positive outcomes they reported were improved service access and increased clinical placement capacity.
*Jones et al. 2015b; Australia	Students have a five‐day intensive induction in Broken Hill, weekly clinical and professional reflection sessions, as well as mid‐ and end of placement focus group evaluations.	Type of sessions is not specified, but they include additional student peer support and learning.	6:1 student to supervisor ratio	Student participation improved continuity of care and capacity to practice inter‐professionally
*Jones et al. 2015c; Australia	Students have a five‐day intensive induction in Broken Hill, weekly clinical and professional reflection sessions, as well as mid‐ and end of placement focus group evaluations.	A combination of face‐to‐face, email, SMS and telephone communications. Supervisions included student peer support and learning as well.	No information reported	Although students initially struggled to transition from being observers to autonomous/semi‐autonomous healthcare providers, they also felt they had real life experience and skills that would help with their future employability.
Jones et al. 2016; Australia	Relevant information was reported in the paper by *Jones et al. (2015)	Relevant information was reported in the paper by *Jones et al. (2015)	Not reported	Authors highlighted the need for and importance of developing community‐literate health students, academics, and practicing professionals, in order for colleges and universities to create a rural‐ready and responsive health workforce.
*Kirby et al. 2018; Australia	No information reported	No information reported	No information reported	The main components helping build partnerships were long‐term work and social relationships, commitment to community, trust and risk‐taking.
Koutsoftas et al. 2024; USA	Students received traditional clinical supervision during speech therapy sessions provided in the therapy room (a separate location from the classroom). They also met with their supervisor 3 times/day to plan and discuss interventions and other responsibilities (assessment, progress monitoring, etc.). Once a week, a clinical adjunct instructor provided onsite supervision and support, and students developed classroom‐based intervention plans in consult with the supervising speech and language pathologist.	Supervisions were conducted face‐to‐face initially, but had to switch to remote instruction, due to the Covid‐19 pandemic.	Each supervising speech and language pathologist was assigned 2‐3 graduate students	The program received positive feedback from all stakeholders, including the students, supervisors, and schoolteachers who worked with the students, and there was an increase in the number of classroom‐based intervention hours for school children receiving speech‐language services.
Marante and Hall‐Mills 2019; USA‐based	Students attend their assigned school for 2–3 full school days every week for a full university semester. They work under the direction of their supervisor, including at least one planning block on their schedules daily to collaborate with their supervisors and other school staff members. Students also have personalised seminar meetings weekly or biweekly with a university instructor who has background in the schools and who communicates regularly with the supervisors.	No information reported	No information reported	This paper discussed the main content areas and minimum practice experiences that should be incorporated in a school‐based placement model and made recommendations on how to best balance the requirements across the graduate curriculum.
*Moran et al. 2024; Australia	Not applicable	Not applicable	Not applicable	This paper explored the implementation of service learning placements in real world settings and the people‐related, partnerships, and place/space influences that enable or hinder their implementation.
